# Higher dose docosahexaenoic acid supplementation during pregnancy and early preterm birth: A randomised, double-blind, adaptive-design superiority trial

**DOI:** 10.1016/j.eclinm.2021.100905

**Published:** 2021-05-17

**Authors:** Susan E Carlson, Byron J Gajewski, Christina J Valentine, Elizabeth H Kerling, Carl P Weiner, Michael Cackovic, Catalin S Buhimschi, Lynette K Rogers, Scott A Sands, Alexandra R Brown, Dinesh Pal Mudaranthakam, Sarah A Crawford, Emily A DeFranco

**Affiliations:** a Department of Dietetics and Nutrition, University of Kansas Medical Center, Kansas City, KS, United States; b Department of Biostatistics & Data Science, University of Kansas Medical Center, Kansas City, KS, United States; c Department of Obstetrics and Gynecology, University of Cincinnati, Cincinnati, OH, United States; d Department of Dietetics and Nutrition, University of Kansas Medical Center, Kansas City, KS, United States; e Department of Obstetrics and Gynecology, University of Kansas Medical Center, Kansas City, KS, United States; f Department of Obstetrics and Gynecology, Ohio State University, Columbus, OH, United States; g Department of Obstetrics and Gynecology, University of Illinois, Chicago, Chicago, IL, United States; h Nationwide Children's Hospital, Columbus, OH, United States; i Department of Dietetics and Nutrition, University of Kansas Medical Center, Kansas City, KS, United States; j Department of Biostatistics & Data Science, University of Kansas Medical Center, Kansas City, KS, United States; k Department of Biostatistics & Data Science, University of Kansas Medical Center, Kansas City, KS, United States; l Department of Dietetics and Nutrition, University of Kansas Medical Center, Kansas City, KS, United States; m Department of Obstetrics and Gynecology, University of Cincinnati, Cincinnati, OH, United States

**Keywords:** Early preterm birth, Gestation less than 34 weeks, Pregnancy, Docosahexaenoic acid (DHA) amount

## Abstract

**Background:**

Several meta analyses have concluded n-3 fatty acids, including docosahexaenoic acid (DHA), reduce early preterm birth (EPB, < 34 weeks), however, the amount of DHA required is unclear. We hypothesized that 1000 mg DHA per day would be superior to 200 mg, the amount in most prenatal supplements.

**Methods:**

This randomised, multicentre, double-blind, adaptive-design, superiority trial was conducted in three USA medical centres. Women with singleton pregnancies and 12 to 20 weeks gestation were eligible. randomization was generated in SAS® by site in blocks of 4. The planned adaptive design periodically generated allocation ratios favoring the better performing dose. Managing study personnel were blind to treatment until 30 days after the last birth. The primary outcome was EPB by dose and by enrolment DHA status (low/high). Bayesian posterior probabilities (pp) were determined for planned efficacy and safety outcomes using intention-to-treat. The study is registered with ClinicalTrials.gov (NCT02626299) and closed to enrolment.

**Findings:**

Eleven hundred participants (1000 mg, *n* = 576; 200 mg, *n* = 524) were enrolled between June 8, 2016 and March 13, 2020 with the last birth September 5, 2020. 1032 (*n* = 540 and *n* = 492) were included in the primary analyses. The higher dose had a lower EPB rate [1.7% (9/540) vs 2.4% (12/492), pp=0.81] especially if participants had low DHA status at enrolment [2.0% (5/249) vs 4.1%, (9/219), pp=0.93]. Participants with high enrolment DHA status did not realize a dose effect [1000 mg: 1.4% (4/289); 200 mg: 1.1% (3/271), pp = 0.57]. The higher dose was associated with fewer serious adverse events (maternal: chorioamnionitis, premature rupture of membranes and pyelonephritis; neonatal: feeding, genitourinary and neurologic problems, all pp>0.90).

**Interpretation:**

Clinicians could consider prescribing 1000 mg DHA daily during pregnancy to reduce EPB in women with low DHA status if they are able to screen for DHA.

**Funding:**

The National Institutes of Health Child Health and Human Development (NICHD) funded the study. Life's DHA™-S oil, DSM Nutritional Products LLC, Switzerland provided all capsules.

**Panel: research in context**

**Evidence before this study**

We searched for multinational research assessing early preterm birth less than 34 weeks in randomized trials of n-3 long chain fatty acid supplementation during pregnancy in PubMed, MEDLINE, Google and ClinicalTrials.gov for articles published between Jan 1, 1990 and Feb 1, 2015 using the terms “DHA” OR “docosahexaenoic acid” OR “n-3 fatty acids” OR “omega-3 fatty acids” AND “pregnancy” OR “preterm birth” without language restrictions. Prior to our study, two meta-analyses had concluded n-3 fatty acids could significantly reduce early preterm birth (birth less than 34 weeks gestation). No prior studies were designed to determine early preterm birth as a primary outcome or examine DHA dose. The “Australian Omega-3 to Reduce the Incidence of Preterm Birth” (ORIP) trial had just begun. Like our trial, “Assessment of DHA on Reducing Early Preterm Birth” (ADORE), ORIP was designed to measure early preterm birth as a primary outcome.

**Added value of this study**

To our knowledge, this is the first to compare 1000 mg DHA to 200 mg DHA, the amount in most prenatal supplements that provide DHA, to prevent early preterm birth < 34 weeks. The study found 1000 mg DHA was likely better than 200 mg DHA in reducing early preterm birth <34 weeks (posterior probability or pp = 0.91), especially for women with low DHA status at the time they were enrolled (pp = 0.93). The higher dose also resulted in fewer maternal and neonatal serious adverse events.

**Implications of all the available evidence**

Clinicians could advise pregnant women with low DHA status to consume a DHA supplement of 1000 mg per day to reduce their odds of delivering before 34 weeks gestation. Our evidence added to evidence from the 2018 Cochrane Review and secondary results from the ORIP trial published in 2020 could be used by the USA to set a Dietary Reference Intake for DHA in pregnancy.

Alt-text: Unlabelled box

## 1 Introduction

Early preterm birth (EPB), defined as birth before 34 weeks gestation [1], is of great concern as these births result in the highest risk of infant mortality, child disability, and societal cost [2]. In the USA, the rate of EPB was 2.75% with the vast majority of preterm births occurring from 34 to 36 weeks of gestation [1]. Emerging evidence from early gestation suggests the risk of spontaneous preterm labor differs mechanistically from term labor [3]. To date, few methods (e.g. cervical length evaluation, maternal biomarkers) have been proposed to predict women at high risk of preterm birth, and few are used clinically. However, even if pregnancies at risk for preterm birth could be identified, reliable prevention measures have been elusive. A recent Cochrane Review concluded there is strong evidence that n-3 long chain polyunsaturated fatty acid supplementation, including docosahexaenoic acid (DHA), can reduce EPBs by nearly half [4]; however, the optimal dose was never identified, the type of omega-3 supplementation was not tested and the analysis included both DHA and eicosapentaenoic acid (EPA) supplementation.

A low dietary intake of DHA is common in parts of the world that consume little or no seafood. Women in the USA consume only about 60 mg dietary DHA daily [5]. In the Kansas University DHA Outcomes Study (KUDOS), conducted from 2006 to 2010, we commonly observed very low DHA status before women were randomised to 0 or 600 mg DHA daily [6]. Their mean red blood cell phospholipid DHA at enrolment was only 4.3% of total fatty acids. In comparison, the mean level reported in pregnant persons in Norway, a country with a very low rate of preterm birth, is 6.9% [7]. Although a primary outcome of the KUDOS was to determine the effect of DHA supplementation on pregnancy outcome in low-risk pregnancies, a secondary finding was a much lower rate of EPB in the supplemented compared to the placebo group (0.6% vs. 4.8%) [6]. That finding led to the study we report here, which included both high- and low-risk pregnancies.

The primary aim of “Assessment of DHA on Reducing Early Preterm Birth” (ADORE) was to determine whether participants assigned to a prenatal supplement of 1000 mg DHA daily would have a lower rate of EPB than those assigned to 200 mg daily. Although, the USA National Academy of Medicine does not set a Dietary Recommended Intake (DRI) for DHA in pregnancy, the FAO/WHO [8] recommends a minimum intake of 200 to 300 mg of DHA per day and up to 1000 mg during pregnancy and lactation. In the past 10 years, 200 mg has become a standard addition to many prenatal supplements. Because prenatal supplements with DHA were already being consumed by some women in the USA when we proposed the trial in 2015, we conducted a superiority trial comparing a dose of 1000 mg to the standard prenatal dose of 200 mg.

## 2 Methods

### 2.1 Study design

This was a multicentre, double-blind, randomised, superiority trial of women recruited at one of three large academic medical centers in the United States (University of Kansas, Ohio State University and University of Cincinnati). The University of Kansas Medical Center granted approval under a central IRB with reliance by the other institutions (STUDY00003455). The study protocol is published [9]. The trial is registered (ClinicalTrials.gov: NCT02626299). Both the study protocol and statistical analysis plan are accessible at https://r2d2.kumc.edu/ADORE/index.jsp. We chose a Bayesian Adaptive Design with efficiency in mind as there is broad acceptance these designs save time and money and lead to more ethical studies [10]. During protocol design, we conducted extensive trial simulations comparing different designs measuring the resources (time and number of patients required) and selected the implemented design as the most effective and efficient. Based on our estimation, the most likely scenario was for 3 and 1% EPB in standard (low) and high dose groups, respectively.

### 2.2 Participants

At enrolment, participants were ≥ 18 years old, 12 to 20 weeks gestation, able to speak and read in either English or Spanish and agreed to consume the capsules provided. Women excluded were those with multifetal gestations, < 18 years old, and < 12 or > 20 weeks gestation, unwilling to discontinue a daily prenatal vitamin DHA supplement of 200 mg or more, or allergic to any component of DHA (including algae) or vegetable oil. The USA Food and Drug Administration (FDA) required DHA to be studied as an Investigational New Drug (IND #129,482), and they imposed limited exclusion criteria to ensure the trial was generalisable. All participants gave written consent.

### 2.3 Randomization and masking

**Fig. 1:**
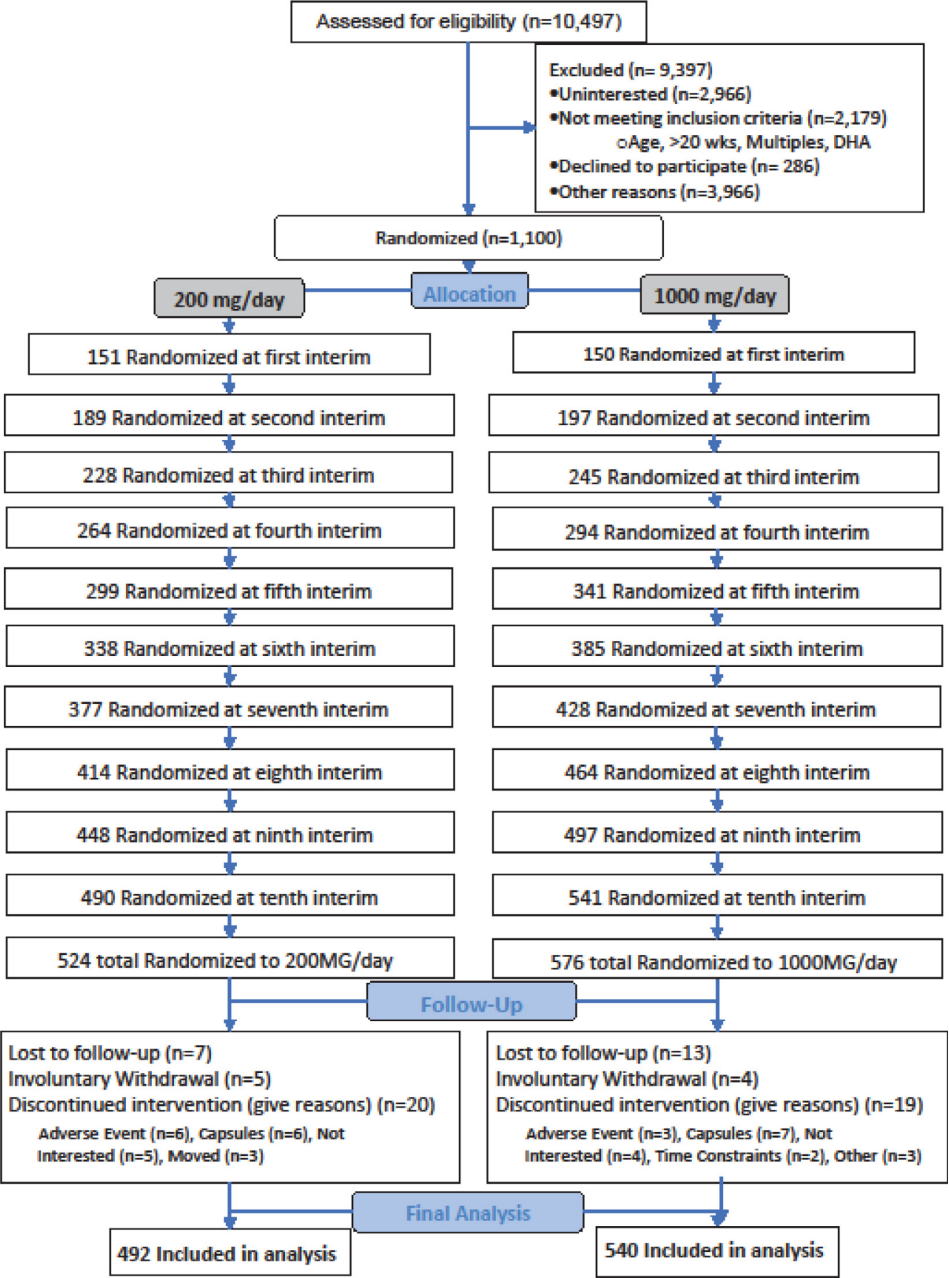
Trial profile.

Participants were randomised to one of two groups (200 or 1000 mg) with a maximum number of pregnant persons n_max_ = 1100 enrolments and 5% expected dropout. Each study site had a separate randomization table to reduce the potential for geographical bias. The initial allocation was in blocks of 4 using an electronic research tool (Powered by WCG Velos, Fremont, CA) customized by the University of Kansas Medical Center Department of Biostatistics & Data Science. Participants gave consent in person, and their data were entered into the electronic research tool. After the participants’ data were entered, the research tool revealed the dose assignment as black stripe or solid black. The assigned bottles of capsules, also coded as black stripe or solid black, were then administered by the study recruiter. Following the planned Bayesian Adaptive Design, after the intial 1:1 allocation, trial data on EPB were used to generate new allocation tables that were appended to the existing tables, a process repeated every 13 weeks until enrolment ended. A total of ten allocations were generated (Fig. 1). All members of the study team and participants were blinded to assignment throughout the trial except two members of the team (ARB, DPM), who were responsible for conducting each interim analysis and adding the new allocation tables. Neither had contact with participants. Capsules were dispensed in opaque bottles.

### 2.4 Procedures

All participants received a bottle containing 200 mg DHA capsules and were instructed to take one capsule daily. Also, participants were randomly assigned to take 2 additional capsules daily that contained either a mixture of corn and soybean oil (low dose) or 400 mg of an algal source of DHA (high dose) (Life's DHA™-S oil, DSM Nutritional Products LLC, Switzerland) for a total possible DHA exposure of 200 or 1000 mg daily. Investigational capsules were orange flavored so participants with eructation could not guess assignment. Study personnel supplied participants the first bottles at enrolment. Thereafter, participants received additional capsules by mail monthly from the University of Cincinnati Investigational Pharmacy and returned unconsumed capsules by mail to the Pharmacy for counting and disposal. At the end of the study, one member of the team (ARB) audited the Investigational Pharmacy refills against the planned allocations and identified two errors. In the first case, an incorrect bottle was mailed the day the participant indicated she had stopped capsule intake. In the second, the participant received the correct treatment from the bottle dispersed at enrolment but the incorrect treatment by mail. Both women were included in the analysis according to their assigned treatment.

After enrolment, study personnel were in monthly contact with participants until delivery to encourage compliance and inquire about any participant concerns. Maternal and umbilical cord bloods were obtained at delivery by medical center clinical staff and trained study personnel prepared samples for storage. Participants who wished to discontinue capsule intake chose in writing whether to allow their blood samples and medical record to be used or to withdraw from the study.

### 2.5 Outcomes

The primary efficacy outcome, the effect of DHA dose on EPB, was centrally assessed. Pregnancy dating was determined in accordance with the American College of Obstetrics and Gynecology guidelines [11] and typically confirmed by an ultrasound at less than 14 weeks gestation. Secondary efficacy outcomes included preterm birth (birth < 37 weeks gestation), maternal DHA status (red blood cell phospholipid DHA as a percent of total fatty acids) at enrolment and delivery, neonate DHA status; very low birth weight (< 1500 g), low birth weight (< 2500 g), gestational age at delivery, weight, length and head circumference at delivery, and pregnancy outcomes (gestational diabetes, preeclampsia, Cesarean delivery, spontaneous or induced labor, and occurrence and reason for non-routine hospitalization).

We recorded serious adverse events (death, life-threatening event, hospital admission, persistent or significant disability/incapacity or congenital anomaly/birth defect) and adverse events (clinical signs and symptoms possibly related to DHA safety). Because our focus was preterm birth, we recorded preterm delivery/preterm birth as both a maternal and neonatal adverse event, while serious maternal and neonatal adverse events related to preterm birth were recorded in the relevant maternal or neonatal disease or organ category.

#### 2.5.1 Blood collection and analysis

Maternal blood samples were collected at enrolment and during the antenatal hospitalization when delivery was imminent, or the following morning if an antenatal sample was not obtained. Maternal blood and umbilical cord blood were collected in EDTA tubes (BD Vacutainer, Franklin Lakes, NJ), placed on wet ice and processed for storage within 24 h. Plasma, buffy coat and red blood cell (RBC) fractions were separated by centrifugation (3000x*g*, 10 min, 4 °C) and stored at −80 °C in barcoded vials before analysis. DHA was determined in maternal samples obtained at enrolment and delivery and on umbilical cord blood (RBC phospholipid weight percent of total fatty acids). All analyses were completed at the University of Kansas Medical Center in the laboratory of a principal investigator (SEC) as described previously [6], with a 10 min transmethylation prior to separation of fatty acid methyl esters by gas chromatography. We considered an enrolment RBC phospholipid DHA < 6% as indicative of low DHA status and a DHA% ≥6% as high status. We chose 6% as a cut off based on a secondary analysis [12] from the Omega-3 to Reduce the Incidence of Preterm Birth (ORIP) trial [13], which showed supplementation reduced EPB only in participants with low DHA status at enrolment. To convert the blood spot procedure used to determine DHA status in ORIP to our red blood cell phospholipid, we divided 2.85%, where the plot in Simmonds et al. [12] changes risk profiles, by 0.4754.

### 2.6 Statistical analysis

The response adaptive randomization allowed for potentially varying sample size if either dose was better performing. The EPB rates by DHA dose drove the randomization probabilities and stopping criteria. EPB rates by dose were modeled as treatment-specific binomial distributions [9]. The planned enrollment was changed twice during the trial. First, we changed from 1355 to 1200, because the expected dropout rate was much lower than expected. Later this was changed to 1100 because of a pause in the enrollment from the pandemic. Both decisions are substantiated by power calculations that are described in the statistical analysis plan (https://r2d2.kumc.edu/ADORE/index.jsp). The interim analyses were prespecified to occur every 13 weeks after 300 participants had been enrolled and until 1100 participants were enrolled. With each interim analysis, an updated randomization schedule was generated. The study would have stopped after 800 participants were enrolled had there been a posterior probability greater than 0.99 of one dose having lower EPB rate than the other. The posterior probability (pp) represents the probability the EPB rate from 1000 mg is lower than that of women given 200 mg. Similarly, the 95% credible interval represents the EPB rate interval having 0.95 probability given the trial data. Bayesian quantities are calculated for secondary outcomes and the safety data. Based on the prespecified design, this study had 80% power with approximately 5% Type I error to detect the best dose [9]. The ADORE trial was powered using a Bayesian model for a dichotomous variable of EPB. In addition, with the availability of new statistical methodology, we utilized a more sophisticated modeling approach that used a mixture of three normal distributions to model gestational age at birth as a continuous time-to-event value, an approach much more efficient than the binomial model [14]. We utilized the continuous data to dichotomise EPB and to model EPB in subgroups with low and high DHA enrolment status. The plan to analyze the interaction between baseline DHA status and dose was added while the trial was ongoing because of new data [12] from the ORIP trial [13].

All the secondary measures used a Bayesian model that was binomial for binary measures and normal for continuous measures. For specific adverse and serious adverse events, we fit a multilevel binomial [15] model by using the incidence of events by body category across doses for both mothers and infants.

All analyses were performed using the intention-to-treat dataset. Data of participants who withdrew or were lost to follow-up were treated as missing and multiple imputation was performed within the Bayesian model. We utilized OpenBUGS version 3.2.3 rev 1012 for all Bayesian analyses. All analyses were fitted using 10,000 burn-in draws of Markov chain Monte Carlo, followed by 40,000 draws for inference.

The study data safety monitoring committee included two neonatologists, one obstetrician and a pediatric epidemiologist, who evaluated progress of the study and adverse events yearly. The trial was registered with ClinicalTrials.gov (NCT02626299) on December 8, 2015.

An exploratory analysis of the recently published ORIP trial concluded that participants who began the study in the highest quartile of n-3 fatty acids status had an *increased risk* of EPB (2.2%) with supplementation relative to participants with high n-3 fatty acid status given the placebo (0.8%) [12]. Because of concern raised by this report, we conducted a similar analysis of women in ADORE by quartile of DHA status at enrolment.

### 2.7 Role of the funding source

The National Institutes of Health Child Health and Human Development (NICHD) funded the study but had no role in study design, data collection, data analysis, data interpretation, or writing of the Article. Life's DHA™-S oil, DSM Nutritional Products LLC, Switzerland provided all capsules, but the company had no role in any aspect of the study or writing of the report. The corresponding author and principal investigators (BJG, CKV) had full access to all the data in the study and had final responsibility for the decision to submit the manuscript.

## 3 Results

Participants were enrolled between June 8, 2016 and March 13, 2020. Out of the 10,497 women screened for eligibility, 9397 were excluded leaving us with 1100 participants who were enrolled under an adaptive design (Fig. 1 trial profile,1000 mg, *n* = 576; 200 mg, *n* = 524). 1032 had observed outcome data (*n* = 540 and *n* = 492). Participant characteristics are included in Table 1**.** Red blood cell phospholipid DHA at enrolment was a mean of 6.4 ± 1.8%. Prior to enrolment, forty-seven percent of participants (491/1032) reported consuming a prenatal supplement of DHA and 40% of those (195/491) consumed a supplement with ≥ 200 mg per day.

**Table 1:** Baseline characteristics of the intention-to-treat population.

**Baseline Characteristic**	**200** **mg/day** ***N*** **=** **524 (47.6%)**	**1000** **mg/day *N*** **=** **576 (52.4%)**	**Total*N*** **=** **1100**
**Site**			
University of Cincinnati Medical center	118 (22.5)	134 (23.3)	252 (22.9)
Ohio State University	173 (33.0)	186 (32.3)	359 (32.64)
University of Kansas Medical center	233 (44.5)	256 (44.4)	489 (44.5)
**Age at enrolment, yr**	30.0 ± 5.8	30.3 ± 5.6	30.2 ± 5.7
	***N*** **=** **522**	***N*** **=** **571**	***N*** **=** **1093**
**DHA mean% of RBC total fatty acids (SD)**^1^	6.46 ± 1.77	6.30 ± 1.76	6.38 ± 1.77
**Pre-pregnancy BMI, kg/m**^2^**, mean (SD)**	28.2 ± 7.3	28.3 ± 7.3	28.2 ± 7.3
**Marital Status, n (%)**			
Married/partnered	335 (63.9)	369 (64.1)	704 (64.0)
Other^2^	189 (36.1)	207 (35.9)	396 (36.0)
**Maternal Race and Ethnicity, n (%)**			
American Indian or Alaskan Native	2 (0.4)	2 (0.4)	4 (0.4)
Asian	9 (1.7)	18 (3.1)	27 (2.5)
Black or African American	122 (23.3)	118 (20.5)	240 (21.8)
Hispanic	109 (20.8)	136 (23.6)	245 (22.3)
Native Hawaiian or Pacific Islander	0 (0.0)	1 (0.2)	1 (0.1)
White	265 (50.6)	288 (50.0)	553 (50.3)
Biracial: Asian, White	6 (1.2)	2 (0.4)	8 (0.7)
Biracial: Asian, Black	0 (0.0)	1 (0.2)	1 (0.1)
Biracial: Black, White	6 (1.2)	5 (0.9)	11 (1.0)
Biracial: Native American, White	0 (0.0)	1 (0.2)	1 (0.1)
Multiracial: Asian, Black, White	1 (0.2)	0 (0.0)	1 (0.1)
Multiracial: Black, Native American, White	0 (0.0)	3 (0.5)	3 (0.3)
Other^3^	4 (0.8)	1 (0.2)	5 (0.5)
**Maternal Education, n (%)**			
Less than high school graduate	69 (13.2)	90 (15.6)	159 (14.5)
HS graduate or equivalent	112 (21.4)	121 (21)	233 (21.2)
Some college or tech school	99 (18.9)	114 (19.8)	213 (19.4)
Bachelor's degree obtained	133 (25.4)	116 (20.1)	249 (22.6)
Master's degree obtained	76 (14.5)	84 (14.6)	160 (14.6)
Doctorate	35 (6.7)	51 (8.9)	86 (7.8)
**Family Income, n (%)**			
Less than $15,000	112 (21.4)	118 (20.5)	230 (20.9)
$15,000 - $24,999	58 (11.1)	78 (13.5)	136 (12.4)
$25,000 - $49,999	92 (17.6)	98 (17)	190 (17.3)
$50,000 - $99,999	93 (17.8)	113 (19.6)	206 (18.7)
$100,000 - $149,999	96 (18.3)	92 (16)	188 (17.1)
At least $150,000	59 (11.3)	59 (10.2)	118 (10.7)
Unknown	14 (2.7)	18 (3.1)	32 (2.9)
**Ever Smoker, yes n (%)**	126 (24.0)	151 (26.2)	277 (25.2)
6 Months prior, yes n (%)	71 (13.5)	71 (12.3)	142 (12.9)
Current smoker, yes n (%)	32 (6.1)	25 (4.3)	57 (5.2)
**Pregnancy History, n (%)**			
Primagravida	160/522 (30.7)	164/572 (28.7)	324/1094 (29.6)
Prior preterm birth^4^	67/362 (18.5)	75/408 (18.4)	142/770 (18.4)
Prior early preterm birth (<34 wks)^4^	27/362 (7.4)	27/408 (6.6)	58/770 (7.0)

1 7 missing baseline blood.

2 divorced (17), refused (2), separated (12), unmarried/single (365).

3 200MG: “Asian-Pakistani”, “Arab”, “Middle Eastern” 1000MG: “Arab, White”.

4 participants with a prior pregnancy.

**Fig. 2:**
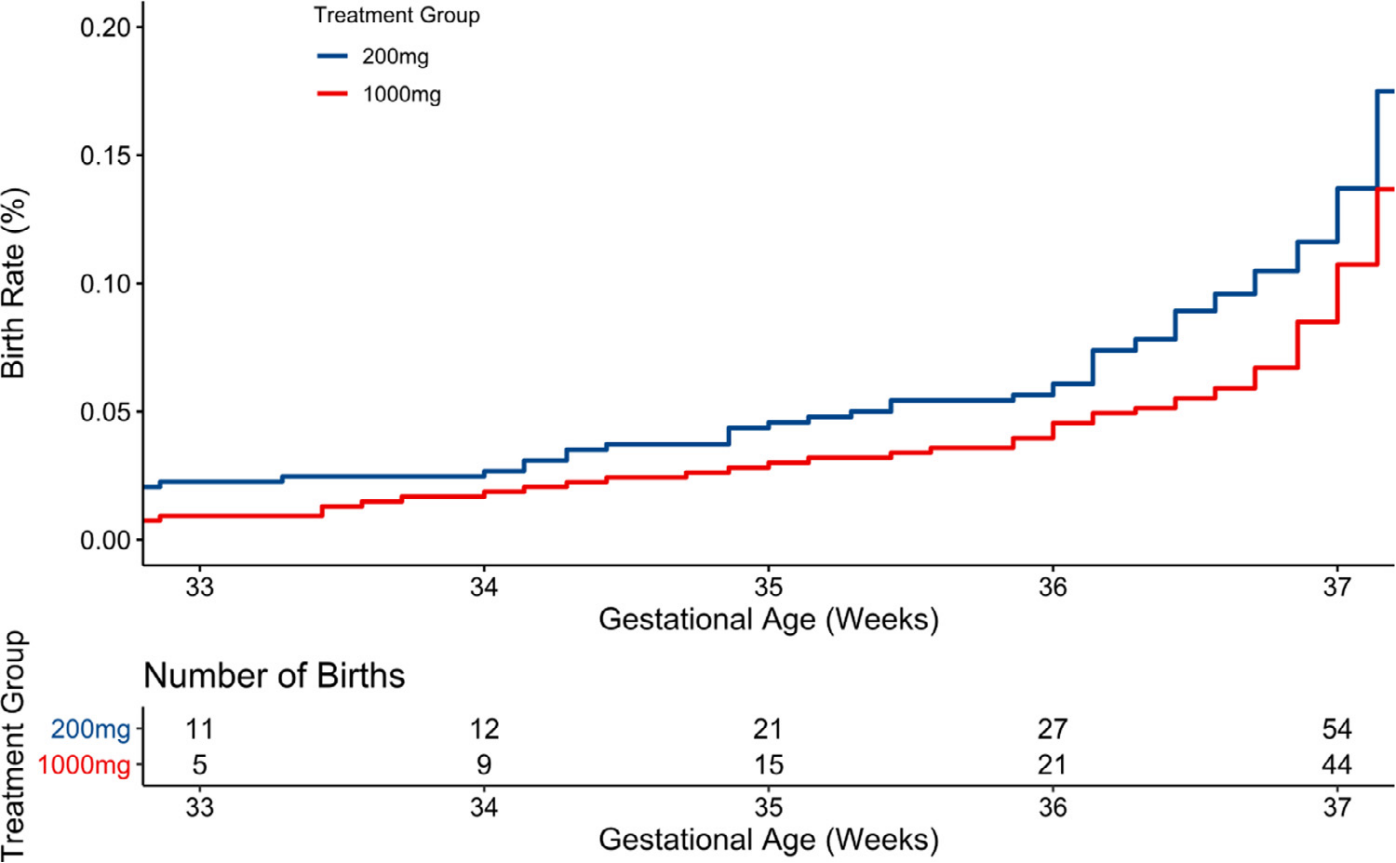
Primary efficacy analysis by group Bayesian posterior probability (pp) =0.91 for less early preterm birth (EPB) with the higher dose. The denominator for the 200 mg dose is 492 and for the 1000 mg dose is 540.

The observed rates of EPB were 1.7% (9/540) and 2.4% (12/492) for the high and low dose, respectively. The binomial analysis (Table 2) indicated a posterior probability (pp) of 0.81 that 1000 mg was better than 200 mg for prevention of EPB. Modeled as time-to-event, the posterior probability means and 95% Bayesian credible interval rates were 1.7% (CI 0.8%, 2.8%) for 1000 mg and 2.9% (CI 1.6%,4.3%) for 200 mg (pp=0.91) **(**Table 2**,** Fig. 2**).** A pp = 0.81 or 0.91 is much larger than an indifferent probability of 0.50 but not as strong as almost certainty (e.g., > 0.99). The number needed to treat may be calculated from Bayesian point estimates for group to be 100/(2.9–1.7) = 83.

**Table 2:** Primary efficacy outcomes.

	**Observed Proportion of Births (%)**		**Posterior mean%** **(95% Bayesian credible interval)**		**Bayesian posterior prob.** (1000 better than 200)
	**200** **mg***N* = 492	**1000** **mg***N* = 540	**200** **mg***N* = 524	**1000** **mg***N* = 576	
**Group**					
Early preterm birth < 34 wk^a^	12/492 (2.4)	9/540 (1.7)	2.5 (1.2, 3.8)	1.7 (0.7, 2.8)	0.81
Early preterm birth < 34 wk^b^	12/492 (2.4)	9/540 (1.7)	2.9 (1.6, 4.3)	1.7 (0.8, 2.8)	0.91
**Subgroups – Birth <34 wk by baseline DHA**^c^					
Low DHA (< 6%) b	9/219 (4.1)	5/249 (2.0)	4.8 (2.3, 7.4)	2.5 (0.8, 4.3)	0.93
High DHA (≥ 6%)^b^	3/271 (1.1)	4/289 (1.4)	1.6 (0.4, 3.0)	1.4 (0.3, 2.7)	0.57

a Uses the primary analysis model that drove the adaptations, which is a Bayesian binomial model.

b Uses an alternative model that dichotomizes the continuous variable via a continuous mixture of three normal distributions.

c Low DHA status at baseline was defined as less than 6% of total red blood cell phospholipid fatty acids.

**Fig. 3:**
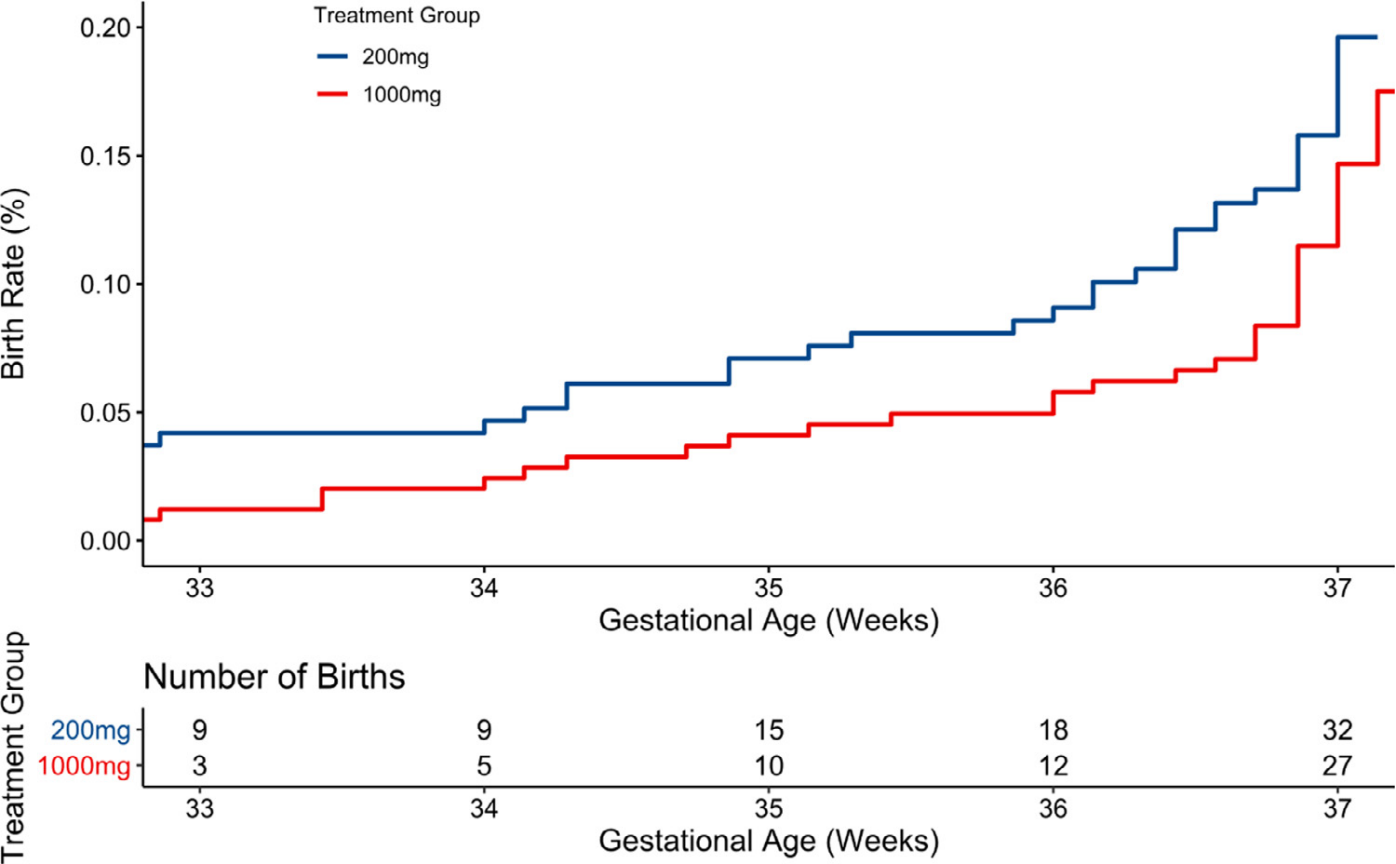
Efficacy analysis in participants with low DHA status at enrolment (red blood cell DHA <6% of total fatty acids) by DHA dose Bayesian posterior probability (pp) = 0.93 for less early preterm birth (EPB) with the higher dose. The denominator for the 200 mg group is 219 and for the 1000 mg group is 249.

Participants enrolling with low DHA status had an observed EPB rate of 2.0% (5/249) if assigned to 1000 mg and 4.1% (9/219) if assigned to 200 mg. Modeled as time-to-event, the posterior mean rates of EPB and 95% Bayesian credible intervals were 2.5% (0.8%,4.3%) and 4.8% (2.3%,7.4%) for the high and low doses, respectively (pp = 0.93) (Table 2, Fig. 3). The number needed to treat may be calculated as 100/(4.8–2.5)=43 for women beginning with low DHA status. In contrast, the EPB rates were low and unaffected by dose in participants who had a high enrolment DHA status. The observed rates for the high and low doses, respectively, were 1.4% (4/289) and 1.1% (3/271). Time-to-event modeling resulted in posterior probability means and credible intervals of 1.4% (0.3%, 2.7%) and 1.6% (0.4%,3.0%) for the high and low doses (pp = 0.57) (Table 2, Supplemental **Fig. 1).** The number needed to treat may be calculated as 100/(1.6–1.4)=500.

The observed outcomes, posterior means and intervals, and Bayesian posterior probabilities for planned secondary outcomes are shown in Table 3. Both maternal postpartum and umbilical cord blood DHA levels at birth had a pp=1.0, confirming the higher dose resulted in higher DHA status. Reported capsule intake is shown in **Supplemental Table 1.** Of the 1032 participants included in the analysis, 102 (9.9%) stopped capsule intake at some point in the study. Participants assigned to the 1000 mg dose also had pp ≥ .90 for longer gestation, greater birth weight, greater birth length, fewer spontaneous labors, fewer preterm births, and fewer neonatal admissions to an intensive care unit. In contrast, women assigned to the lower dose had a pp ≥ .87 for less preeclampsia and gestational diabetes.

**Table 3:** Secondary efficacy outcomes.

	**Observed Outcome of Births**		**Posterior mean** **(95% Bayesian credible interval)**		**Bayesian posterior prob.** (1000 better than 200)
	**200 mg** *N* = 492	**1000 mg** *N* = 540	**200 mg** *N* = 524	**1000 mg** *N* = 576	
					
Birth weight (g), mean (std)	*N* = 489 3264 (574)	*N* = 539 3326 (542)	3264 (3215, 3314)	3327 (3279, 3374)	0.96
Birth length (cm), mean (std)	*N* = 487 49.9 (4.4)	*N* = 529 50.2 (3.6)	49.9 (49.5, 50.2)	50.2 (49.9, 50.5)	0.90
Head circumference (cm), mean (std)	*N* = 480 33.9 (2.3)	*N* = 526 33.9 (2.5)	33.9 (33.7, 34.1)	33.9 (33.7, 34.1)	0.49
Preterm birth (<37 weeks), %^a^	54/492 (11.0)	44/540 (8.2)	13.1% (10.8%, 15.5%)	10.5% (8.5%, 12.5%)	0.95
Very low birth weight (< 1500 g),%^a^	7/489 (1.4)	4/539 (0.7)	0.7% (0.0%, 1.3%)	0.5% (0.0%, 1.1%)	0.80
Low birth weight (< 2500 g),% ^a^	36/489 (7.4)	27/539 (5.0)	6.8% (4.9%, 9.1%)	5.4% (3.9%, 7.3%)	0.87
Gestation age birth (days), mean (std)	269.9 (16.3)	271.8 (11.1)	270 (269, 271)	272 (271, 273)	0.99
Maternal RBC DHA% by weight, mean (std)	*N* = 441 7.6 (2.2)	*N* = 475 9.9 (3.5)	7.7 (7.4, 7.9)	10.0 (9.7, 10.2)	1.00
Cord RBC DHA% by weight, mean (std)	*N* = 439 8.9 (2.1)	*N* = 476 10.0 (2.5)	8.9 (8.7, 9.1)	10.0 (9.8, 10.2)	1.00
Gestational diabetes, %	48/489 (9.8)	68/540 (12.6)	10.1% (7.5%, 12.9%)	12.8% (10.1%, 15.8%)	0.08
Preeclampsia, %	27/489 (5.5)	39/539 (7.2)	5.7% (3.7%, 8.0%)	7.3% (5.2%, 9.8%)	0.13
Cesarean delivery, %	137/492 (27.9)	144/540 (26.7)	27.9% (24.0%, 32.0%)	26.8% (23.1%, 30.6%)	0.65
Spontaneous labor, %	143/492 (29.1)	142/540 (26.3)	29.1% (25.1%, 33.3%)	26.4% (22.7%, 30.2%)	0.16
Intensive care admission, %	58/492 (11.8)	50/540 (9.3)	11.9% (9.2%, 15.0%)	9.4% (7.1%, 12.0%)	0.91

All models are Binomial (flat priors) or Normal (flat priors) with posterior means and 95% highest density intervals unless notated. At least 10,000 burn-in and 40,000 Markov chain draws were performed. ^a^ Uses an alternative model that dichotomizes the continuous variable via a continuous mixture of three normal distributions.

**Supplemental Table 2** shows the rates of combined adverse or serious adverse events by dose in mothers and neonates. The higher dose was favored for fewer maternal (pp = 0.89) and neonatal (pp = 0.94) adverse and serious adverse events. Specific adverse and serious adverse events for mothers are shown in **Supplemental Table 3** and for neonates in **Supplemental Table 4**. Participants assigned to the higher dose had pp > 0.90 for fewer maternal adverse and serious adverse events related to premature rupture of membranes and pyelonephritis; fewer serious adverse events due to chorioamnionitis; and fewer adverse events of weight loss, preterm birth and preterm contractions. Infants of mothers assigned to the higher dose had a pp > 0.90 for fewer adverse and serious events related to feeding, fewer serious events related to genitourinary and neurologic symptoms, and fewer adverse events of preterm birth and musculoskeletal problems. The low dose did not favor a reduction in any type of maternal or infant serious adverse event but it did favor fewer skin adverse events.

Because premature rupture of membranes and chorioamnionitis are common causes of early labor while preeclampsia may lead to preterm birth without labor, we looked at these outcomes in our EPBs. Among women assigned to the low dose, 83% (10/12) of EPBs were spontaneous while 17% (2/12) were associated with provider initiated preterm birth in the setting of preeclampsia. In contrast, 22% (2/9) of EPBs in the high dose group were due to early spontaneous labor. Of the remaining seven participants who had an EPB, one participant did not take capsules and six had preeclampsia.

An exploratory analysis of the recently published ORIP trial found that participants who began the study in the highest quartile of n-3 fatty acids had an *increased risk* of EPB (2.2%) with supplementation relative to participants with high n-3 fatty acids who received the placebo (0.8%) [12]. Because of concern raised by this report, we conducted a similar analysis of the participants in ADORE by quartile of DHA status at enrolment. In contrast to ORIP, participants who began the study in the highest quartile for DHA status had an exceedingly low rate of EPB regardless of dose [200 mg: 0/131 (0%); 1000 mg: 1/131 (0.8%)]. Participants in the lower two DHA quartiles at enrolment benefited from the higher dose with a lower rate of EPB, but there was no benefit of a higher dose for women in the higher two quartiles (**Supplemental Table 5**).

## 4 Discussion

The results of this randomised clinical trial show that a daily supplement of 1000 mg DHA is likely better than 200 mg in reducing EPB less than 34 weeks (pp =0.91), and very likely better among pregnant individuals with low DHA status (pp = 0.93). Participants entering the study with low DHA status had half the rate of EPB when assigned to the high compared to the low dose. Participants who began the study with high DHA status had a very low rate of EPB (1.2%), and the rate was not different between DHA doses.

Because by study design we did not have a placebo group, we cannot conclude that women entering the study with high DHA status benefited from either dose. On the other hand, most participants in the subgroup who entered with high DHA status were taking a DHA-containing prenatal supplement before they were enrolled, so we also cannot conclude that DHA supplementation did not reduce their rate of EPB. Low dose supplementation beginning early and maintained throughout pregnancy may have a ceiling effect, providing enough DHA to reduce EPB. Only 2% of the women in ADORE delivered before 34 weeks in contrast to a rate of 7.0% in those with prior pregnancies. The very high historical rate may reflect the fact that we were encouraged by the FDA to enroll women regardless of pregnancy risk. The large difference suggests that even the low dose may have had a benefit, but it could also be related to the fact that participants who qualified for progesterone and cerclage under the American College of Obstetrics and Gynecology guidelines received these treatments intended to prevent preterm birth.

At the time this trial was proposed, four randomised, placebo-controlled trials that provided a dietary supplement of DHA ≥ 600 mg had been reported [6,[16], [17], [18]] .Of these, three found a reduction in EPB [6,16,17] as a secondary outcome. In addition, two published systematic reviews that included all randomised trials regardless of the amount of n-3 fatty acid supplementation during pregnancy found odds ratios favoring supplementation of 0.69 [19] and 0.74 [20]. An updated Cochrane review published several years after our trial was proposed included all placebo controlled pregnancy trials of n-3 fatty acid supplementation regardless of n-3 fatty acid source and found an OR = 0.58 [4] for a reduction in EPB. This review concluded that low doses of n-3 fatty acids do not reduce EPB. The review also indicated there was no effect of DHA on birth < 37 weeks when dosed at less than 500 mg per day. Because of the findings from this review, we chose to use 1000 mg per day and a microalgal oil as our DHA supplement to harmonize with our earlier trial [6].

The study was powered using simulations from the results of the two trials that had been conducted in singleton pregnancies [6,16]. We chose a superiority rather than a placebo-controlled trial because FAO/WHO and other expert groups were already recommending pregnant persons consume a minimum of 200 to 300 mg DHA per day. Based on this guidance, manufacturers had added 200 mg DHA to many prenatal supplements beginning around 2008, and an increasing number of women in the USA were consuming a prenatal supplement with DHA despite the absence of evidence that this amount of DHA can reduce EPB.

Our finding that women beginning the trial with low DHA status benefited most from the high dose of DHA agrees with exploratory findings from the ORIP trial [12]. The ORIP investigators observed that singleton pregnancies with low n-3 fatty acid status at enrolment benefited with n-3 fatty acids supplementation (~800 mg DHA) (i.e., lower rate of EPB less than 34 weeks) compared to placebo (0.73% vs 3.16%) [12] even though the primary trial found no significant effect of supplementation [13]. Women in ADORE who enrolled with a low DHA status similar to low status in ORIP had EPB rates of 2.0% and 4.1%, in the high and low dose groups, respectively. While our rates are slightly higher than reported for ORIP [12], Black women comprised 22% of our cohort and in the USA have higher rates of EPB compared to women of other races and ethnicities [1].

Controversy has arisen following an exploratory analysis of the ORIP trial, which suggested that supplementing women with already high n-3 fatty acid status could increase rather than decrease their risk of EPB [13]. We addressed this safety concern with a similar analysis in the ADORE cohort and found no evidence of increased risk. On the contrary, our results show a progressive decline in EPB from the lowest to the highest quartile of DHA status at enrolment (3.6%, 2.8%, 1.5% and 0.4%, respectively).

While the mechanism of preterm birth remains elusive and complex [21], biomarkers assessed prior to 20 weeks gestation suggest spontaneous preterm labor may be associated with stressors linked to inflammation [3,[22], [23], [24]]. DHA is a precursor of docosanoids (resolvins, maresins) that are anti-inflammatory and that resolve and protect against inflammation [25], and both resolvin D1 and D2 from DHA and inflammatory mediators formed from the n-6 fatty acid, arachidonic acid, increase with spontaneous preterm birth [26]. We observed fewer cases of spontaneous preterm birth, which are known to be driven by inflammatory mechanisms [22], [23], [24], in the high compared to the low DHA dose group. Because of this, we suggest that our finding of lower EPB with higher DHA supplementation could be due to a change in balance between n-3 and n-6 derived mediators to reduce or prevent the inflammatory process associated with labor.

The most recent Cochrane Review supports implementation of DHA supplementation in pregnancy at a dose between 500 and 1000 mg per day [4]. The exploratory results of the Australian ORIP trial [12] and those of ADORE provide evidence that DHA supplementation needs to be higher than in current prenatal supplements for women with low DHA status. Future directions should include educating physicians and women about the importance of consuming more DHA during pregnancy, which could be achieved by a combination of seafood consumption and supplements that contain DHA. Supplement options include the microalgal oil as provided in ADORE as well as fish oils that contain DHA. Future studies should refine the optimal dose and the best time during pregnancy to increase DHA intake. Studies of a dose intermediate between 200 and 1000 mg and of women supplemented early in or prior to pregnancy are needed. Studies to improve DHA status before pregnancy are also needed as a lower dose may be effective if provided earlier. While we don't have a clear explanation for the higher probability of gestational diabetes mellitus and preeclampsia in the 1000 mg compared to the 200 mg dose, it is possible that prolongation of pregnancy allowed for development and diagnosis of these two conditions at more advanced gestational ages. Gestational diabetes was highest in our Hispanic participants and preeclampsia in our Black participants compared to other racial/ethnic categories. Both groups were oversampled in the ADORE cohort. While our safety data overall favor the higher dose, future studies should continue to monitor for risk.

The few exclusion criteria and oversampling of Black and Hispanic women, who are at higher risk of preterm birth compared to other race/ethnic groups, are strengths of the study. We also obtained results from 95% of the women enrolled and documented their DHA status at enrolment and delivery. The intention-to-treat analysis reported here likely underestimates the true benefit of DHA to reduce EPB, because not all women were fully compliant with their assigned capsule intake. We plan to report the per protocol analysis later. Another limitation of the study is that there were fewer EPBs than anticipated. As already noted, women who qualified for progesterone and cerclage under the American College of Obstetrics and Gynecology guidelines received those treatments intended to prevent preterm birth (5.5% and 5.0% in the low and high dose groups, respectively). At the time this study was planned and conducted, the EPB rate in the USA was 3.4% and 7% for non-Black and Black women [27]. The most recent data show 2.3% and 4.9%, respectively [1], possibly reflecting the fact that many US women now consume a low dose supplement of DHA during pregnancy.

The findings here, combined with the most recent Cochrane Review and the results of ORIP, have implications for both policy and practice. Regarding policy, the National Academy of Medicine in the USA could now set a Dietary Recommended Intake for DHA in pregnancy, not possible before an amount of DHA could be linked to lower EPB. Regarding clinical practice, clinicians could consider testing DHA status and offering high dose DHA supplementation to those with low DHA status. Women with high DHA status at enrolment should be encouraged to take a prenatal with 200 mg DHA. The routine ability to determine DHA status is currently a limitation. We point out that pregnancy studies use plasma [28] and whole blood spot DHA [13] as well as RBC phospholipid DHA to measure DHA status. While DHA status is highly correlated among methods, DHA will be a lower percentage of total fatty acids in plasma and whole blood compared to RBC phospholipids. We include a factor to convert RBC phospholipids to whole blood spot DHA. North America and Africa have the highest rates of preterm birth, however, the burden of preterm birth in numbers disproportionately occurs in Africa and Asia [29], where many countries have low DHA intake and status [30]. Easy access to an inexpensive test of DHA status or a simple tool screener for DHA intake could help clinicians identify women most likely to benefit from a higher dose.

## Declaration of Competing Interest

None.

## Appendix Supplementary materials

Image, application 1
