# Supplementary material for: Higher dose docosahexaenoic acid supplementation during pregnancy and early preterm birth: A randomised, double-blind, adaptive-design superiority trial
Source: eClinicalMedicine. 2021 May 17;36:100905. doi: 10.1016/j.eclinm.2021.100905 (PMC8257993; doi:10.1016/j.eclinm.2021.100905)
Supplement: Supplementary file 1 [file mmc1.docx]

**Caption for supplementary material**

**Supplemental Table 1: Daily capsule count intake reported on study**

**Supplemental Table 2: Overall rate of maternal and neonatal adverse and serious adverse events**

**Supplemental Table 3: Maternal Adverse and Serious Adverse Events**

**Supplemental Table 4: Neonatal Adverse and Serious Adverse Events**

**Supplemental Table 5: Observed early preterm birth rate by dose and quartile DHA status at enrolment and posterior means, Bayesian 95% credible intervals and posterior probabilities of a dose effect**

**Supplemental Figure 1: Efficacy analysis in participants enrolling with high DHA status (red blood cell phospholipid DHA ≥6% of total fatty acids) by DHA dose**

|  |  | **200 mg/day**  **N=524** | **1000 mg/day N=576** | **Total**  **N=1100** |
| --- | --- | --- | --- | --- |
| **Capsules consumed, mean (SD)** |  |  |  |  |
| Labelled DHA (200 MG) |  | n=488 0.8 (0.4) | n=533 0.8 (0.4) | n=1021 0.8 (0.4) |
| Investigational supplement^1^ (0 or 800 MG) |  | n=452 1.5 (0.8) | n=491 1.5 (0.7) | n=943 1.5 (0.7) |
| ^1^The 200 mg/day group received two placebo capsules in the investigational supplement daily. The 1000 mg/day received two capsules with 400 mg DHA daily in the investigational supplement. | | | | |

**Supplemental Table 1: Daily capsule count intake reported on study**

|  | | | | | |
| --- | --- | --- | --- | --- | --- |
|  | **Count of Events (%)** | | **Posterior mean % (95% Bayesian credible interval)** | | **Bayesian posterior prob.** (1000 better  than 200) |
|  | **200 mg** | **1000 mg** | **200 mg** | **1000 mg** |  |
|  |  |  |  |  |  |
| **Mothers** | N=524 | N=576 |  |  |  |
| Adverse events | 422 (80.5) | 446 (77.4) | 80.5 (77.0, 83.8) | 77.4 (73.9, 80.8) | 0.89 |
| Serious adverse events | 71 (13.5) | 64 (11.1) | 13.5 (10.7, 16.6) | 11.1 (8.6, 13.9) | 0.89 |
|  |  |  |  |  |  |
| **Infants** | N=492 | N=540 |  |  |  |
| Adverse events | 139 (28.3) | 116 (21.5) | 28.3 (24.2, 32.3) | 21.5 (18.1, 25.1) | 0.99 |
| Serious adverse events | 65 (13.2) | 54 (10.0) | 13.2 (10.3, 16.3) | 10.0 (7.6, 12.7) | 0.94 |
| All models use Bayesian binomial model with flat priors. | | | | | |
|  | | | | | |

**Supplemental Table 2: Overall rate of maternal and neonatal adverse and serious adverse events**

|  | | | | | | |
| --- | --- | --- | --- | --- | --- | --- |
|  | **Adverse Events**  **No. of Mothers (%)** | | **Bayesian posterior prob.** (1000 better than 200) | **Serious Adverse Events**  **No. of Mothers (%)** | | **Bayesian posterior prob.** (1000 better than 200) |
|  | **200 mg**  N = 524 | **1000 mg**  N = 576 |  | **200 mg**  N = 524 | **1000 mg**  N = 576 |  |
| **Cardiovascular/Blood** |  |  |  |  |  |  |
| Arrhythmia^1^ | 1 (0.2) | 0 (0) | 0.72 | 1 (0.2) | 0 (0) | 0.80 |
| Decreased hemoglobin/anemia | 217 (41.4) | 243 (42.2) | 0.37 | 0 (0) | 0 (0) | 0.76 |
| Increased BP^1^ | 24 (4.6) | 31 (5.4) | 0.29 | 4 (0.8) | 5 (0.9) | 0.51 |
| Other AEs^1^ | 11 (2.1) | 12 (2.1) | 0.52 | 4 (0.8) | 2 (0.3) | 0.77 |
| Pre-eclampsia | 30 (5.7) | 42 (7.3) | 0.17 | 14 (2.7) | 19 (3.3) | 0.29 |
| **Eyes, Ears, Nose & Throat** |  |  |  |  |  |  |
| Blurred vision^1^ | 8 (1.5) | 4 (0.7) | 0.88 | 0 (0) | 0 (0) | 0.87 |
| Nasal congestion^1^ | 3 (0.6) | 4 (0.7) | 0.69 | 0 (0) | 0 (0) | 0.87 |
| Other AEs | 10 (1.9) | 5 (0.9) | 0.89 | 1 (0.2) | 0 (0) | 0.88 |
| Sinus drainage^1^ | 2 (0.4) | 0 (0) | **0.93** | 0 (0) | 0 (0) | 0.87 |
| Sinusitis^1^ | 1 (0.2) | 3 (0.5) | 0.68 | 0 (0) | 0 (0) | 0.87 |
| **Gastrointestinal** |  |  |  |  |  |  |
| Diarrhea | 30 (5.7) | 27 (4.7) | 0.80 | 0 (0) | 0 (0) | 0.83 |
| Emesis | 70 (13.4) | 56 (9.7) | **0.96** | 1 (0.4) | 1 (0.2) | 0.73 |
| Heartburn/acid reflux | 83 (15.8) | 89 (15.5) | 0.56 | 0 (0) | 0 (0) | 0.83 |
| Nausea | 90 (17.2) | 91 (15.8) | 0.71 | 0 (0) | 0 (0) | 0.83 |
| Other AEs^1^ | 19 (3.6) | 20 (3.5) | 0.66 | 1 (0.2) | 1 (0.2) | 0.73 |
| Stomach Pain | 11 (2.1) | 11 (1.9) | 0.73 | 1 (0.2) | 0 (0) | 0.84 |
| **Head, Neck & Mental Wellbeing** |  |  |  |  |  |  |
| Anxiety^1^ | 0 (0) | 2 (0.3) | 0.57 | 0 (0) | 0 (0) | 0.84 |
| Depression^1^ | 4 (0.8) | 7 (1.2) | 0.39 | 1 (0.2) | 0 (0) | 0.86 |
| Headache^1^ | 28 (5.3) | 34 (5.9) | 0.29 | 2 (0.4) | 1 (0.2) | 0.78 |
| Other AEs^1^ | 7 (1.3) | 3 (0.5) | 0.85 | 1 (0.2) | 1 (0.2) | 0.75 |
| **Metabolic and Nutrition** |  |  |  |  |  |  |
| Abnormal 1hr OGTT^1^ | 11 (2.1) | 8 (1.4) | 0.77 | 0 (0) | 0 (0) | 0.71 |
| Gestational diabetes mellitus^1^ | 10 (1.9) | 16 (2.8) | 0.22 | 0 (0) | 3 (0.5) | 0.26 |
| Hypoglycemia^1^ | 1 (0.2) | 0 (0) | 0.89 | 0 (0) | 0 (0) | 0.71 |
| Other AEs^1^ | 4 (0.8) | 5 (0.9) | 0.61 | 1 (0.2) | 1 (0.2) | 0.57 |
| Weight loss^1^ | 4 (0.8) | 1 (0.2) | **0.91** | 0 (0) | 0 (0) | 0.71 |
| **Other** |  |  |  |  |  |  |
| Unable to categorize^1^ | 2 (0.4) | 2 (0.3) | 0.72 | 0 (0) | 0 (0) | 0.82 |
| **Pregnancy/Delivery** |  |  |  |  |  |  |
| Chorioamnionitis | 15 (2.9) | 12 (2.1) | 0.80 | 2 (0.4) | 0 (0) | **0.90** |
| Decreased fetal movement | 43 (8.2) | 58 (10.1) | 0.19 | 2 (0.4) | 1 (0.2) | 0.83 |
| Endometritis^1^ | 2 (0.4) | 2 (0.3) | 0.72 | 2 (0.4) | 2 (0.3) | 0.74 |
| Hepatobiliary^1^ | 0 (0) | 4 (0.7) | 0.43 | 0 (0) | 0 (0) | 0.84 |
| Laceration (vaginal, introital)^1^ | 15 (2.9) | 15 (2.6) | 0.66 | 0 (0) | 0 (0) | 0.84 |
| Miscarriage | 2 (0.4) | 1 (0.2) | 0.78 | 2 (0.4) | 1 (0.2) | 0.83 |
| Oligohydramnios | 13 (2.5) | 10 (1.7) | 0.81 | 0 (0) | 0 (0) | 0.84 |
| Other PD AEs^1^ | 0 (0) | 0 (0) | 0.77 | 0 (0) | 0 (0) | 0.84 |
| Placental abnormalities^2^ | 15 (2.9) | 20 (3.5) | 0.41 | 1 (0.2) | 5 (0.9) | 0.39 |
| Polyhydramnios | 23 (4.4) | 19 (3.3) | 0.82 | 1 (0.2) | 0 (0) | 0.87 |
| Post or intra - partum hemorrhage | 22 (4.2) | 22 (3.8) | 0.66 | 0 (0) | 2 (0.3) | 0.61 |
| Premature delivery | 54 (10.3) | 44 (7.6) | **0.93** | 0 (0) | 0 (0) | 0.84 |
| Premature membrane rupture | 27 (5.2) | 16 (2.8) | **0.96** | 16 (3.1) | 7 (1.2) | **0.97** |
| Preterm contractions | 71 (13.5) | 58 (10.1) | **0.95** | 8 (1.5) | 5 (0.9) | 0.85 |
| Rule/out^1^ | 2 (0.4) | 2 (0.3) | 0.71 | 1 (0.2) | 0 (0) | 0.87 |
| Short cervix^1^ | 2 (0.4) | 4 (0.7) | 0.57 | 0 (0) | 0 (0) | 0.84 |
| **Respiratory (RESP)** |  |  |  |  |  |  |
| Bronchitis^1^ | 3 (0.6) | 2 (0.3) | 0.75 | 0 (0) | 0 (0) | 0.79 |
| Cough^1^ | 2 (0.4) | 4 (0.7) | 0.51 | 0 (0) | 0 (0) | 0.79 |
| **Continued on next page** |  |  |  |  |  |  |
| Other AEs^1^ | 7 (1.3) | 7 (1.2) | 0.51 | 2 (0.4) | 2 (0.3) | 0.59 |
| Shortness of breath^1^ | 5 (1) | 7 (1.2) | 0.41 | 1 (0.2) | 2 (0.3) | 0.55 |
| URI | 3 (0.6) | 3 (0.5) | 0.66 | 1 (0.2) | 0 (0) | 0.80 |
| **SKIN** |  |  |  |  |  |  |
| Acne^1^ | 0 (0) | 0 (0) | 0.76 | 0 (0) | 0 (0) | 0.84 |
| General rash^1^ | 4 (0.8) | 4 (0.7) | 0.51 | 0 (0) | 0 (0) | 0.84 |
| MRSA^1^ | 1 (0.2) | 0 (0) | 0.80 | 0 (0) | 0 (0) | 0.84 |
| Other AEs^1^ | 2 (0.4) | 10 (1.7) | **0.05** | 0 (0) | 0 (0) | 0.84 |
| **Urogenital (UG)** |  |  |  |  |  |  |
| Bacterial vaginosis^1^ | 5 (1) | 5 (0.9) | 0.73 | 0 (0) | 0 (0) | 0.83 |
| Other AEs^1^ | 13 (2.5) | 15 (2.6) | 0.54 | 1 (0.2) | 2 (0.3) | 0.62 |
| Pyelonephritis^1^ | 7 (1.3) | 1 (0.2) | **0.97** | 7 (1.3) | 1 (0.2) | **0.94** |
| Sexually transmitted infection^1^ | 2 (0.4) | 2 (0.3) | 0.80 | 0 (0) | 0 (0) | 0.83 |
| Spotting | 24 (4.6) | 21 (3.6) | 0.76 | 0 (0) | 0 (0) | 0.82 |
| Urinary tract infection^1^ | 7 (1.3) | 3 (0.5) | **0.91** | 1 (0.2) | 1 (0.2) | 0.74 |
| Vaginal bleeding | 32 (6.1) | 30 (5.2) | 0.71 | 0 (0) | 2 (0.3) | 0.56 |
| Vaginal discharge^1^ | 11 (2.1) | 17 (3) | 0.31 | 0 (0) | 0 (0) | 0.83 |
| Vaginitis^1^ | 0 (0) | 2 (0.3) | 0.70 | 0 (0) | 0 (0) | 0.83 |
| Yeast infection^1^ | 6 (1.1) | 2 (0.3) | **0.93** | 0 (0) | 0 (0) | 0.83 |
| **Whole Body** |  |  |  |  |  |  |
| Abdominal pain^1^ | 22 (4.2) | 23 (4) | 0.57 | 2 (0.4) | 1 (0.2) | 0.78 |
| Back pain^1^ | 18 (3.4) | 21 (3.6) | 0.46 | 0 (0) | 0 (0) | 0.83 |
| Cramping^1^ | 15 (2.9) | 15 (2.6) | 0.61 | 0 (0) | 0 (0) | 0.83 |
| Fatigue^1^ | 1 (0.2) | 1 (0.2) | 0.74 | 0 (0) | 0 (0) | 0.83 |
| Fever^1^ | 2 (0.4) | 6 (1) | 0.40 | 1 (0.2) | 0 (0) | 0.85 |
| Infection^1^ | 4 (0.8) | 2 (0.3) | 0.81 | 2 (0.4) | 1 (0.2) | 0.78 |
| Lightheadedness^1^ | 4 (0.8) | 3 (0.5) | 0.75 | 0 (0) | 0 (0) | 0.83 |
| Other AEs^1^ | 33 (6.3) | 26 (4.5) | 0.87 | 4 (0.8) | 4 (0.7) | 0.58 |
| Pelvic pain | 53 (10.1) | 58 (10.1) | 0.48 | 0 (0) | 1 (0.2) | 0.71 |
| Swelling/edema^1^ | 10 (1.9) | 14 (2.4) | 0.39 | 0 (0) | 0 (0) | 0.83 |
| Syncope^1^ | 5 (1) | 3 (0.5) | 0.80 | 0 (0) | 0 (0) | 0.83 |
|  |  |  |  |  |  |  |
| ^1^After the first year, these AEs were not recorded from the medical record unless they were serious. ^2^Abnormalities included early diagnostic notching, abruption, previa, circumvallate, placental lake. | | | | | | |
|  | | | | | | |

**Supplemental Table 3: Maternal Adverse and Serious Adverse Events**

|  | | | | | | |
| --- | --- | --- | --- | --- | --- | --- |
|  | **Adverse Events**  **No. of Infants (%)** | | **Bayesian posterior prob.** (1000 better than 200) | **Serious Adverse Events**  **No. of Infants (%)** | | **Bayesian posterior prob.** (1000 better than 200) |
|  | **200 mg**  N = 492 | **1000 mg**  N = 540 |  | **200 mg**  N = 492 | **1000 mg**  N = 540 |  |
| **Infant Adverse Event** |  |  |  |  |  |  |
| Birth asphyxia | 0 (0) | 0 (0) | 0.83 | 0 (0) | 0 (0) | 0.85 |
| Cardiology/cardiac | 8 (1.6) | 7 (1.3) | 0.77 | 4 (0.8) | 2 (0.4) | 0.86 |
| Congenital anomaly | 6 (1.2) | 4 (0.7) | 0.84 | 4 (0.8) | 3 (0.6) | 0.80 |
| Dermatology | 3 (0.6) | 2 (0.4) | 0.82 | 1 (0.2) | 1 (0.2) | 0.81 |
| Endocrine/metabolic | 8 (1.6) | 8 (1.5) | 0.71 | 7 (1.4) | 7 (1.3) | 0.69 |
| Feeding | 14 (2.8) | 7 (1.3) | **0.94** | 12 (2.4) | 6 (1.1) | **0.93** |
| Gastrointestinal | 2 (0.4) | 1 (0.2) | 0.84 | 1 (0.2) | 0 (0) | 0.88 |
| Genitourinary | 6 (1.2) | 3 (0.6) | 0.88 | 2 (0.4) | 0 (0) | **0.91** |
| Head/neck | 8 (1.6) | 7 (1.3) | 0.76 | 0 (0) | 1 (0.2) | 0.77 |
| Hematology/hemorrhage | 1 (0.2) | 6 (1.1) | 0.41 | 0 (0) | 1 (0.2) | 0.77 |
| Hepatobiliary | 12 (2.4) | 13 (2.4) | 0.64 | 4 (0.8) | 3 (0.6) | 0.80 |
| Infection | 12 (2.4) | 12 (2.2) | 0.69 | 4 (0.8) | 3 (0.6) | 0.81 |
| Intrauterine growth restriction | 19 (3.9) | 21 (3.9) | 0.59 | 1 (0.2) | 1 (0.2) | 0.81 |
| Musculoskeletal | 7 (1.4) | 3 (0.6) | **0.91** | 2 (0.6) | 1 (0.2) | 0.85 |
| Neonatal abstinence syndrome | 1 (0.2) | 4 (0.7) | 0.56 | 1 (0.2) | 4 (0.7) | 0.54 |
| Neurologic symptoms | 2 (0.4) | 0 (0) | 0.89 | 2 (0.4) | 0 (0) | **0.91** |
| Preterm birth | 54 (11) | 44 (8.1) | **0.93** | 0 (0) | 0 (0) | 0.86 |
| Respiratory distress | 37 (7.5) | 38 (7) | 0.65 | 24 (4.9) | 28 (5.2) | 0.47 |
| Intrauterine/fetal demise | 4 (0.8) | 2 (0.4) | 0.86 | 4 (0.8) | 2 (0.4) | 0.86 |
| Other infant adverse events^a^ | 0 (0) | 0 (0) | 0.84 | 0 (0) | 0 (0) | 0.86 |
| ^a^ Infant adverse events that could not be categorized into one of the previous event types. | | | | | | |
|  | | | | | | |

**Supplemental Table 4: Neonatal Adverse and Serious Adverse Events**

|  | **Early preterm birth <34 weeks, no./N (%)** | | **Posterior mean%  (95% Bayesian credible interval)^a^** | | **Bayesian posterior prob.** (1000 better than 200) |
| --- | --- | --- | --- | --- | --- |
|  | **200 mg** | **1000 mg** | **200 mg**  N = 524 | **1000 mg**  N = 576 |  |
|  |  |  |  |  |  |
| **DHA quartile at Enrolment** | |  |  |  |  |
| Quartile 1 (0 to 5.06%) | 5/111 (4.5) | 4/140 (2.9) | 3.7 (1.4,7.8) | 1.9 (0.8,3.6) | 0.86 |
| Quartile 2 (5.07 to 6.17%) | 6/129 (4.7) | 1/125 (0.8) | 3.8 (1.6,7.8) | 1.6 (0.5,2.8) | 0.92 |
| Quartile 3 (6.18 to 7.43%) | 1/119 (0.8) | 3/142 (2.1) | 1.5 (0.1,4.0) | 1.8 (0.7,3.1) | 0.37 |
| Quartile 4 (> 7.43%) | 0/131 (0) | 1/131 (0.8) | 1.1 (0.0,3.7) | 1.6 (0.5,2.8) | 0.30 |
| ^a^ Bayesian hierarchical model was fitted using the approach in Berry SM et al,^28^ except  mu~N(-3.1,10^2).This approach avoids false discoveries in subgroups (quartiles). We burned-in 10,000 draws and used 40,000 draws for inference.  [28] Berry SM, Borglio KR, Groshen S, Berry DA. Bayesian hierarchical modeling of patient subpopulations: efficient designs of phase II oncology clinical trials. Clin Trials 2013;10(5):720–34. | | | | | |

**Supplemental Table 5: Observed early preterm birth rate by dose and quartile DHA status at enrolment and posterior means, Bayesian 95% credible intervals and posterior probabilities of a dose effect**


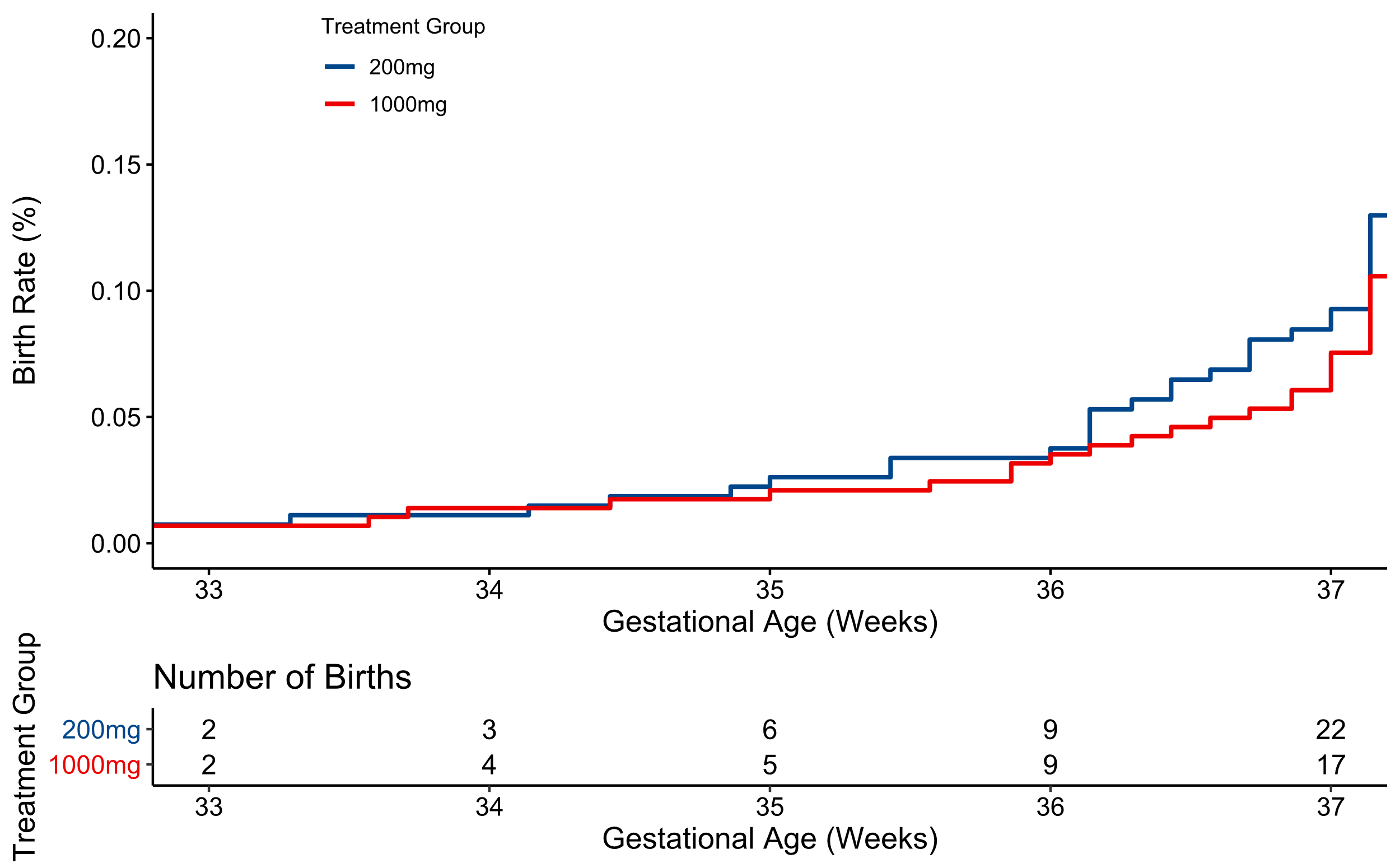


**Supplemental Figure 1: Efficacy analysis in participants enrolling with high DHA status (red blood cell phospholipid DHA ≥6% of total fatty acids) by DHA dose**

Bayesian posterior probability (pp) = .57 indicating it is unlikely doses are different with regard to early preterm birth (EPB). The denominator for the 200 mg group is 271 and for the 1000 mg group is 289.
